# The Physicians’ Visiting List

**Published:** 1899-12

**Authors:** 


THE PHYSICIANS' VISITING LIST for 1900, published by the well-known house of P. Blakiston's Son & Co., has made its annual visit. Like all of its predecessors it is an exceedingly neat and handy little volume, and its arrangement for physicians is all that could be desired. It can easily be carried in the pocket, and is, therefore, a most valuable day-book for the busy practitioner. R.

